# Identifying Pathways for Improving Household Food Self-Sufficiency Outcomes in the Hills of Nepal

**DOI:** 10.1371/journal.pone.0127513

**Published:** 2015-06-05

**Authors:** Tika B. Karki, Shrawan K. Sah, Resam B. Thapa, Andrew J. McDonald, Adam S. Davis

**Affiliations:** 1 Tribhuvan University, Institute of Agriculture and Animal Sciences, Rampur, Nepal; 2 CIMMYT, South Asia Regional Office, Kathmandu, Nepal; 3 United States Department of Agriculture/ Agricultural Research Service, Urbana, Illinois, United States of America; California State University, UNITED STATES

## Abstract

Maintaining and improving household food self-sufficiency (FSS) in mountain regions is an ongoing challenge. There are many facets to the issue, including comparatively high levels of land fragmentation, challenging terrain and transportation bottlenecks, declining labor availability due to out-migration, and low technical knowledge, among others. Using a nonparametric multivariate approach, we quantified primary associations underlying current levels of FSS in the mid-hills of Nepal. A needs assessment survey was administered to 77 households in Lungaun (Baglung District), Pang (Parbat District), and Pathlekhet (Myagdi District), with a total of 80 variables covering five performance areas; resulting data were analyzed using Classification and Regression Trees. The most parsimonious statistical model for household FSS highlighted associations with agronomic management, including yields of maize and fingermillet within a relay cropping system and adoption of improved crop cultivars. Secondary analyses of the variables retained in the first model again focused primarily on crop and livestock management. It thus appears that continued emphasis on technical agricultural improvements is warranted, independent of factors such as land holding size that, in any case, are very difficult to change through development interventions. Initiatives to increase household FSS in the mid-hills of Nepal will benefit from placing a primary focus on methods of agricultural intensification to improve crop yields and effective technology transfer to increase adoption of these methods.

## Introduction

Global inequality in food security is high and continues to grow [1, 2], with recurring food surpluses in economic powerhouses and food deficits in developing regions. There is a broad consensus that food aid is merely palliative [3], and not only does little to address hunger’s root causes, but may actually exacerbate food insecurity. Identifying the origins of food insecurity is a pressing research topic at a global scale, yet one that almost always has context-specific solutions. Addressing hunger in mountain regions is one such example. Mountain areas in many parts of the world face an ongoing struggle to feed their inhabitants [4, 5, 6]. Such environments are often challenging places to produce food due to factors such as altitude, extreme climates, shallow and erosion-prone soils, sloping lands and distance from population centers. Here, we take a quantitative approach to identifying factors that contribute to observed variations in household food self-sufficiency. We define FSS as food produced at the household level, not including purchased food, in the mid-hills of Nepal.

In contrast to Nepal’s foothills and low-lying Terai and inner Terai regions, which are contiguous with the Indo-Gangetic Plain and nearly as fertile, the country’s hill areas face many limits to crop productivity. The mid-hills, which range approximately between 1000 and 2000 m above sea level, are home to almost half of Nepal’s population and a disproportionate share of its hunger [7]. Maize (*Zea mays* L.) and fingermillet (*Eleusine coracana* Gaertin) are the second and fourth most important cereal crops in Nepal, both in terms of area cultivated and total production [8], and are the primary staples in the mid-hills. Hill farmers typically grow these crops in a maize-fingermillet relay cropping system, with fingermillet seedlings transplanted into standing maize crops with varying degrees of temporal overlap depending upon the altitude at which the crops are grown [9]. Land holdings of villagers are small, averaging 0.7 ha per household, and maize and fingermillet grown on these small fields yield only two and one t ha^-1^, respectively, far below their yield potential [10]. Although most households are engaged in farming, 27 out of 75 districts, mainly in the hills and mountains of the mid- and far-western regions, are food-insecure [8].

Achieving and maintaining FSS is a multidimensional problem, depending on a wide range of factors, including agricultural production and distribution, labor availability, land tenure and access to appropriate technology [4, 11, 12]. Declining soil fertility, limited water, trade-offs between grain and forage production, labor shortages, lack of appropriate mechanization, high cost of production and lack of farmer knowledge of best management practices are major limitations to productivity of maize-based systems in mid-hills [13, 14, 15]. Moreover, the persistent outmigration of labor and the need for additional cash remittances to offset declining crop yields and labor inputs often forms a pernicious negative cycle [12].

The objective of our study was to determine which factors, along the performance dimensions mentioned above, appear to be most tightly linked with currently observed variation in household FSS. Given scarce time and resources, and the previous success of stakeholder-driven research in improving food security [16], we addressed this objective with a needs survey administered to small-holders in the mid-hills.

## Materials and Methods

### Description of study area

**Fig 1:**
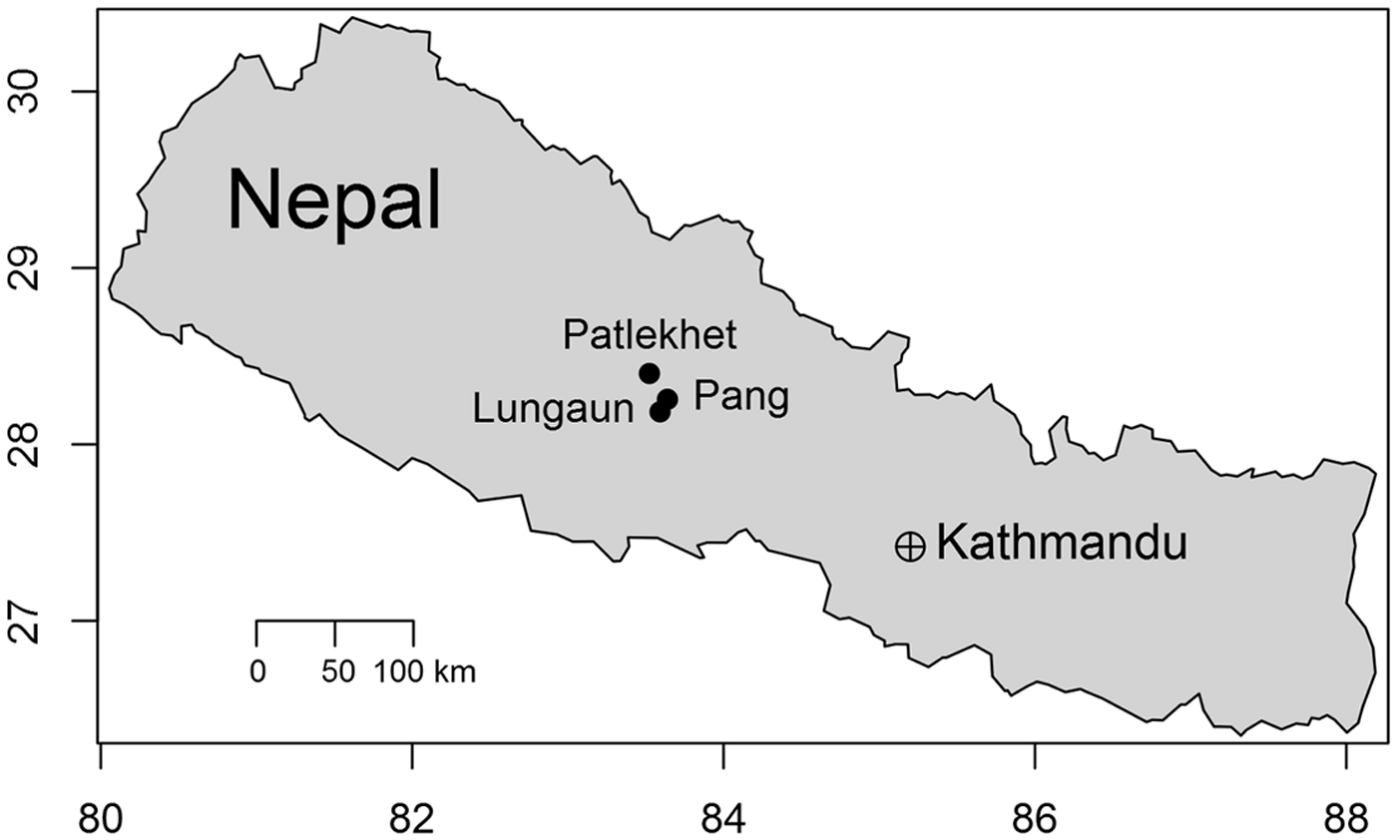
Location of survey districts within Nepal.

A needs assessment survey was conducted in summer 2010 of smallholder farmers in the Western Development Region of Nepal. Three villages in that region, namely Pang, Lungaun and Patlekhet, were randomly chosen as survey locations (Table 1; Fig 1) from a larger candidate pool of villages within three contiguous districts in the western region, Parbat, Baglung and Myagdi. All villages in this candidate pool were selected based on the absence of external governmental and non-governmental agricultural interventions. We reasoned that, in the absence of external development programs, the analysis would emphasize locally-relevant management practices and technologies that at least some farmers in the target region are able to sustainably deploy. These villages also feature maize-based cropping systems across an elevation gradient.

**Table 1:** Survey area description.

Village	District	Latitude (°N)	Longitude (°W)	Elevation range (m)	Major ethnicity	Soil type	Households (No. of households)	Sample size (N)
Pang	Parbat	28.25	83.63	1000–1240	Brahmin, Chhetri	silty-clay	75	25
Lungaun	Baglung	28.18	83.59	1180–1300	Magar	sandy-clay	55	23
Patlekhet	Myagdi	28.40	83.52	1000–1570	Magar, Chhetri	clay-loam	90	27

Pang is home to 75 households, with an average household size of 4.7, Lungaun has 55 households and an average household size of 4.5, and Patlekhet has 90 households, and an average household size of 4.5 as of 2008.

The survey area represents a humid sub-tropical climate, with a unimodal rainfall distribution driven by the summer monsoon. This area received 487 mm rainfall during 2010, the survey year. The highest rainfall was recorded in July (168 mm) followed by June (156 mm). November and January were the driest months, with no rainfall at all. In 2010, the average maximum and minimum temperatures in the region were 22.4° and 12.1°C, respectively.

Soils in these villages are highly weathered, and because the terrain is steep, terraced fields are a common means of slowing overland flow of water and reducing erosion during the wet season. Mechanization and external inputs remain low, with animal traction the primary means of propelling farm implements. Although improved crop varieties have been developed for hill regions of Nepal [10], adoption lags behind variety development and planting open-pollinated land races of food crops remains the norm.

### Survey instrument

**Fig 2:**
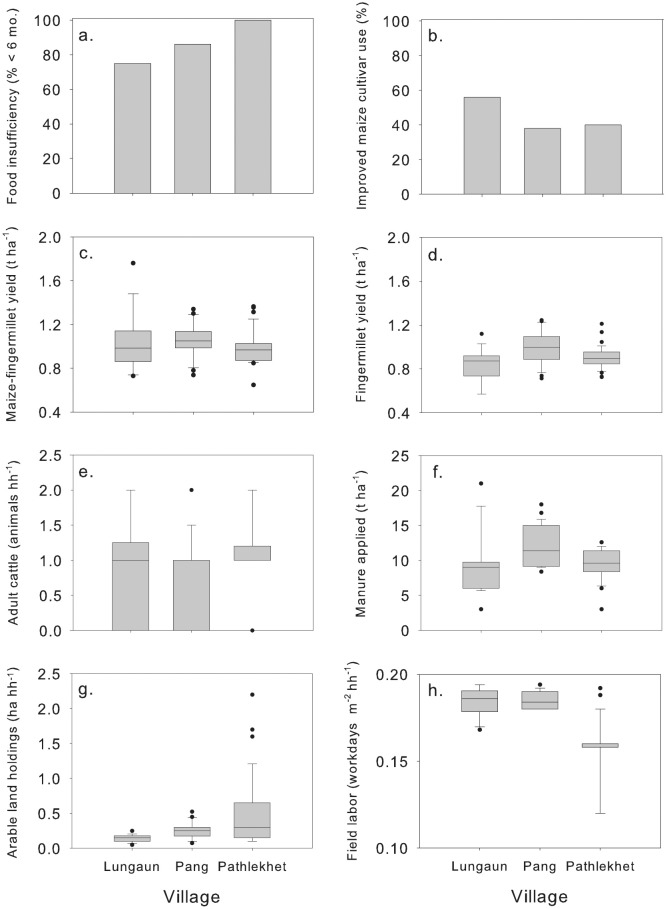
Univariate overview of survey areas. Selected variables include data on household food self-sufficiency, crop and livestock production, land holdings and labor for three villages in the mid-hills of Nepal. Shaded areas in box plots represent interquartile range, interior horizontal line represents the median, and black dots represent extreme values.

The survey consisted of a semi-structured questionnaire, with a total of 80 questions covering five areas. These subject areas included respondent socioeconomic characteristics, land holdings and tenure arrangements, labor availability, crop production and animal husbandry. Socioeconomic information included degree of FSS based on farm production (< 6 months, or 6 to 12 months), literacy, gender and ethnicity. Land holding and tenure variables included altitude, area of irrigated (*Khet*) and un-irrigated (*Bari*) arable land, grassland (*Kharbari*) and woodlot area, number of separate parcels of land owned, land rental agreements, tenancy and sharecropping arrangements. Labor information included the amount of labor available during critical field operations, the amount of labor required m^-2^ on a given homestead, local wages for men, women and children, and labor sharing among neighbors. Crop production variables included farmer-reported yields of sole-crop maize, maize and fingermillet within a maize-fingermillet relay system, wheat, soybean and rice, average productivity of all staple crops, chemical fertilizer use, farmyard manure use, trends in input applications over the past five years, timing of fertilizer application, use of crop and weed residue as forage, and weed management practices. Animal husbandry included number of young and adult cattle, buffalo and goats, and animal management (exclusive stall-fed system, semistall-fed system, free grazing). The entire list of variables, including means and standard errors for quantitative variables and number of observations in different categorical levels for qualitative variables, can be found in S1 Text. Means and standard errors for FSS, yields of the primary crops, animal and land holdings, and labor availability are shown in Fig 2.

### Survey administration

We worked with local Village Development Committees (VDC) to locate and contact respondents. Survey sample size in each of the villages was based on local population density, with the aim of interviewing enough respondents in each village to achieve a 5% margin of error at a 95% confidence level [17]. This condition was satisfied in all locations, with the survey administered to 25, 23 and 29 respondents (overall N = 77) from Pang, Lungaun, and Patlekhet, respectively (Table 1). We met with 2–3 farmers a day, in interviews that generally lasted three hours.

### Ethics statement

This study was approved by the Nepalese Agricultural Research Council (NARC) and Tribhuvan University, Rampur, Nepal. Oral consent was obtained from all participants prior to initiation of the study; as not all subjects were literate, consent was documented in written form by the survey administrator in the presence of each participant. This consent procedure was used after ethical review and approval by Tribhuvan University and NARC. All survey data were analyzed anonymously.

### Data analysis

We used classification and regression trees (CART) [18] to highlight the primary associations between dependent variables of interest (FSS, crop yield and adoption of improved cultivars) and independent variables belonging to the five subject areas included in the survey (socioeconomic, land tenure, labor, crop production and animal husbandry). The use of CART in analyzing complex ecological data sets has become increasingly common due to the ability of this method to describe patterns and processes for a wide range of data types, including numerical and categorical data, with output that is simple to understand [19]. The basic algorithm underlying CART is to repeatedly partition a data set into more and more homogeneous groups, evaluating the relative impurity of the data before and after a split to determine whether added model complexity aids in reducing proportional error [18]. We fit our CART models using 50-fold cross-validation in the ‘rpart’ package of R statistical computing software version 2.15.2 [20]. Our approach to identifying the most parsimonious models for a given dependent variable was to follow the ‘1-se’ rule [21], in which the simplest model within one standard error of the minimum relative model error was considered to provide the best fit to the data with the least number of splits. Output was represented graphically as a dichotomous tree.

Data analysis proceeded in multiple, nested stages. Once the most parsimonious tree was identified for FSS as a dependent variable, we treated each of the variables retained in this analysis as a dependent variable in subsequent, separate CART analyses.

## Results

### Hunger in the mid-hills

The majority of respondents (more than 75%) reported households with low FSS, producing less than six months of food supply and needing to make up this shortfall either through remittances from family working abroad or off-farm labor. Household FSS was the lowest in Lungaun (75% food insecure), intermediate in Pang (86% food insecure) and greatest in Pathlekhet (100% food insecure) (Fig 2a). Maize-finger millet relay cropping systems, with fallow during the winter period, were found to be the major cropping pattern comprising 85% in Parbat, 79% in Baglung and 55% in Myagdi. Adoption of improved maize varieties was greater in Lungaun (57%) than in Pang (39%) or Pathlekhet (37%) (Fig 2b). Reported yields of maize within a maize-fingermillet relay system, and fingermillet, were consistently low (Fig 2c and 2d), averaging 1.0 and 0.9 t ha^-1^, respectively. Most households owned one adult cow (Fig 2e), and applied farmyard manure to fields at a mean rate of 10.3 t ha^-1^ (Fig 2f). Arable land holdings averaged less than a 0.33 ha (Fig 2g), with less than 0.20 workdays m^-2^ of labor available throughout the growing season (Fig 2h).

### CART analysis of household food self-sufficiency

**Fig 3:**
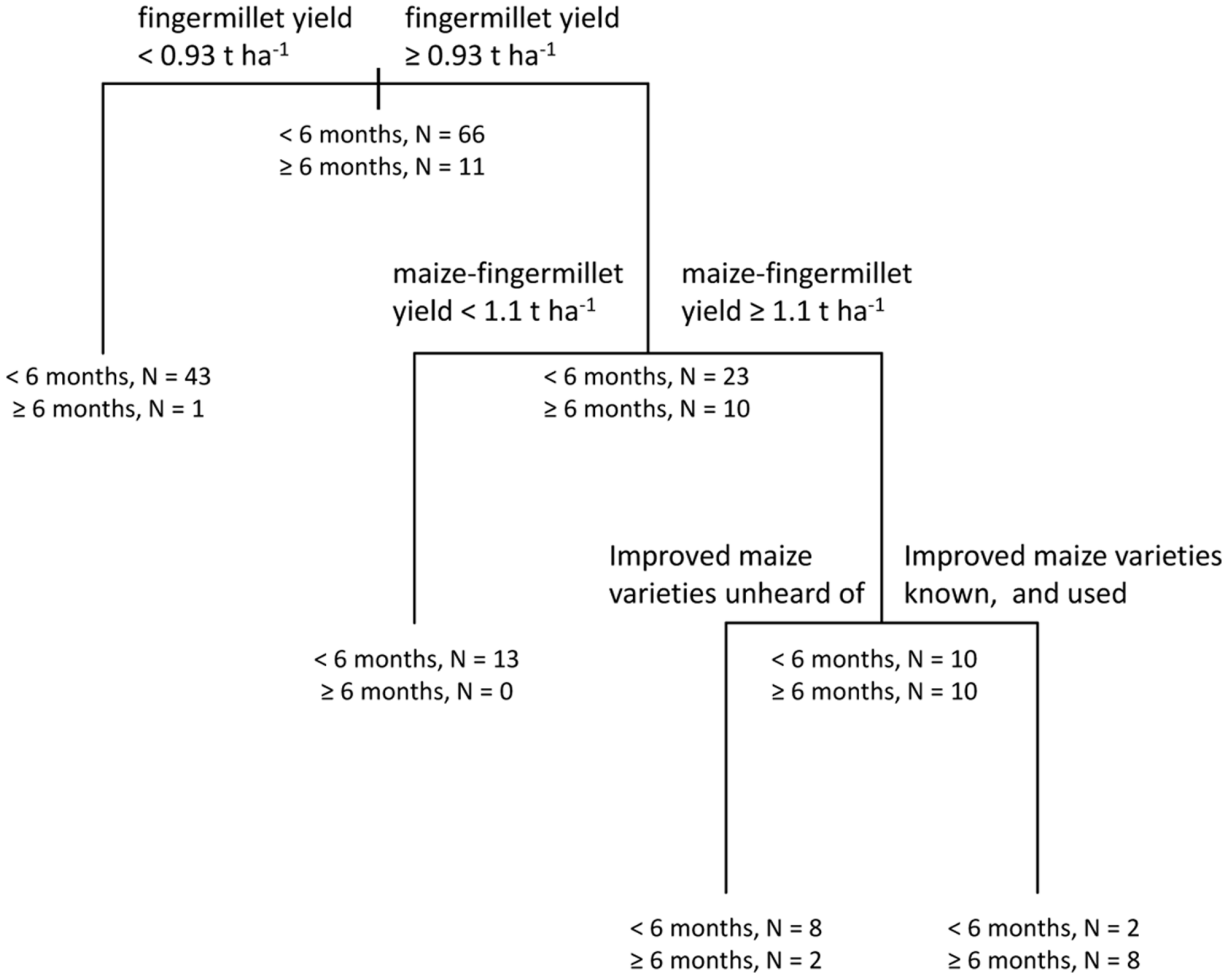
Food self-sufficiency. Classification and regression tree shows associations between the dependent variable ‘food self-sufficiency’ and independent variables retained in the most parsimonious model.

Strong associations were observed between agronomic factors and FSS in the CART model best supported by the data (Fig 3), which explained more than half of the surveyed variation (R^2^ = 0.55). At the root node of the tree, 86% (66 out of 77) of respondents reported having less than six months of FSS. The first branch point occurred at a fingermillet yield of 0.93 t ha^-1^. Of those respondents producing less than this yield benchmark, 99% had less than 6 months FSS, whereas only 70% of respondents achieving or exceeding this benchmark had less than 6 months FSS. The second branch was conditioned by the yield of maize in a maize-fingermillet relay system; 100% of the respondents reporting less than 1.1 t ha^-1^ of maize yield had less than 6 months FSS, compared to 50% of those with yields greater than or equal to that breakpoint. The third branch was associated with reasons given by respondents for use or lack of use of improved maize varieties. Among those who had not heard of improved maize varieties (reflecting access to agricultural knowledge), 80% had less than 6 months of FSS, whereas for those respondents who had heard of, and adopted, improved maize varieties, only 20% had less than six months of FSS. Following the model selection of this initial tree for FSS, we analyzed each of the variables retained in separate CART model selection processes.

#### Fingermillet yield

**Fig 4:**
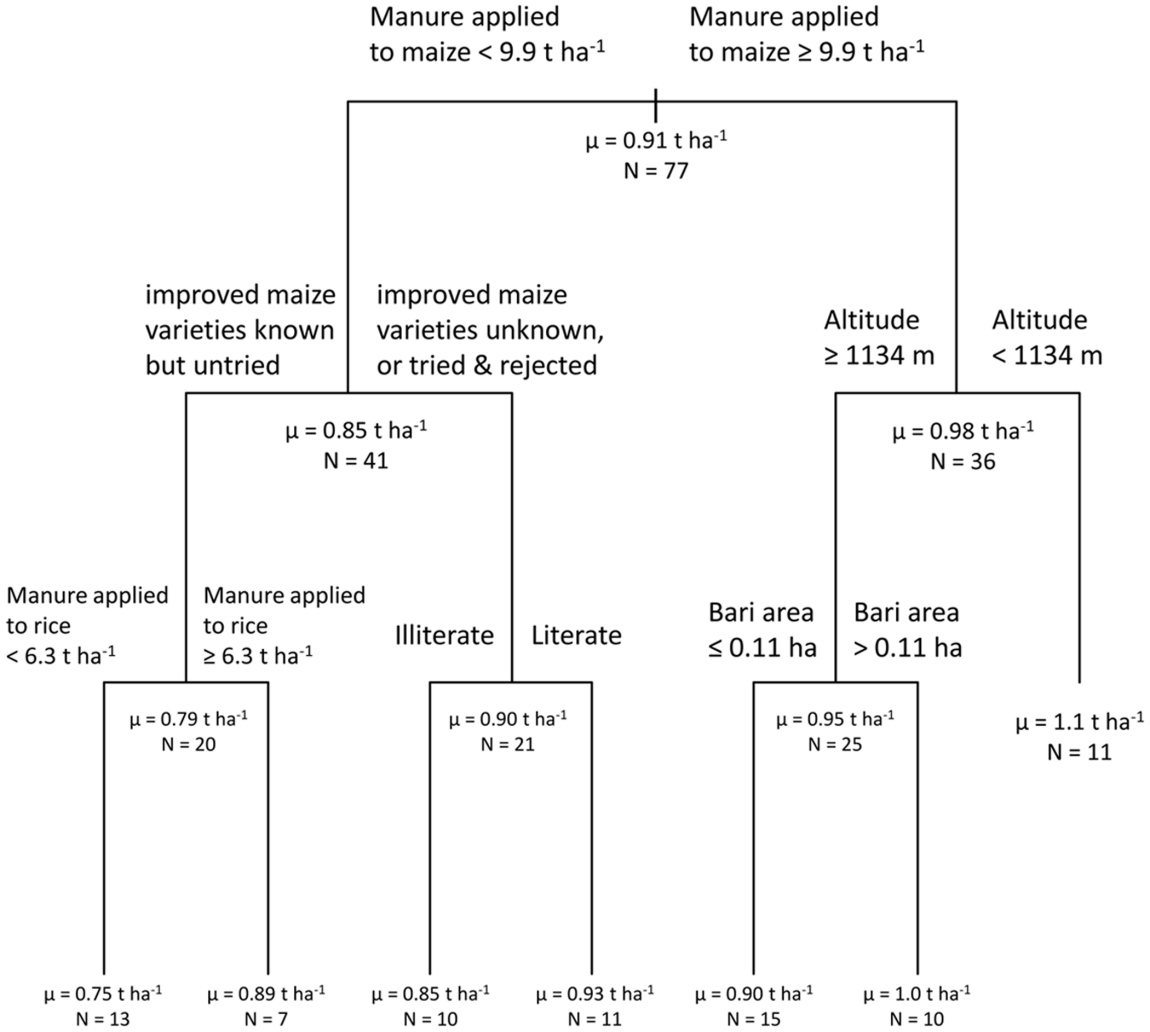
Fingermillet yield. Classification and regression tree shows associations between the dependent variable ‘fingermillet yield’ and independent variables retained in the most parsimonious model.

The most parsimonious CART model for fingermillet yield (Fig 4) explained half of the surveyed variation for this variable (R^2^ = 0.50). Three of the six model nodes pertained to agronomic management (manure application rate to maize and rice, adoption of improved maize varieties), and two other dimensions were represented in the remaining nodes [socioeconomic (literacy) and land holding characteristics (altitude, area of arable land)]. Mean fingermillet yield at the base node was 0.91 t ha^-1^. The initial branch was based on application rates of farmyard manure to maize in the maize-fingermillet relay system, with application rates less than 9.9 t ha^-1^ linked to a mean fingermillet yield of 0.85 t ha^-1^, and application rates greater than or equal to this amount associated with a mean fingermillet yield of 0.98 t ha^-1^. For subsequent branches arising from the first node on the left branch, illiteracy, low manure application rates and hesitance to try improved varieties, despite knowing about their existence, were all associated with lower fingermillet yields. Although manure application to rice fields is not directly linked to fingermillet yields, since the two crops are produced in different types of fields (irrigated and non-irrigated, respectively), the association of higher manure application rates in rice with higher fingermillet yields may simply indicate an overall greater supply of farmyard manure for a given farming operation, and higher soil fertility levels in general. In secondary and tertiary branches arising from the first node on the right branch, lower fingermillet yields were associated with smaller production fields at high altitudes.

#### Maize yield in a maize-fingermillet relay system

**Fig 5:**
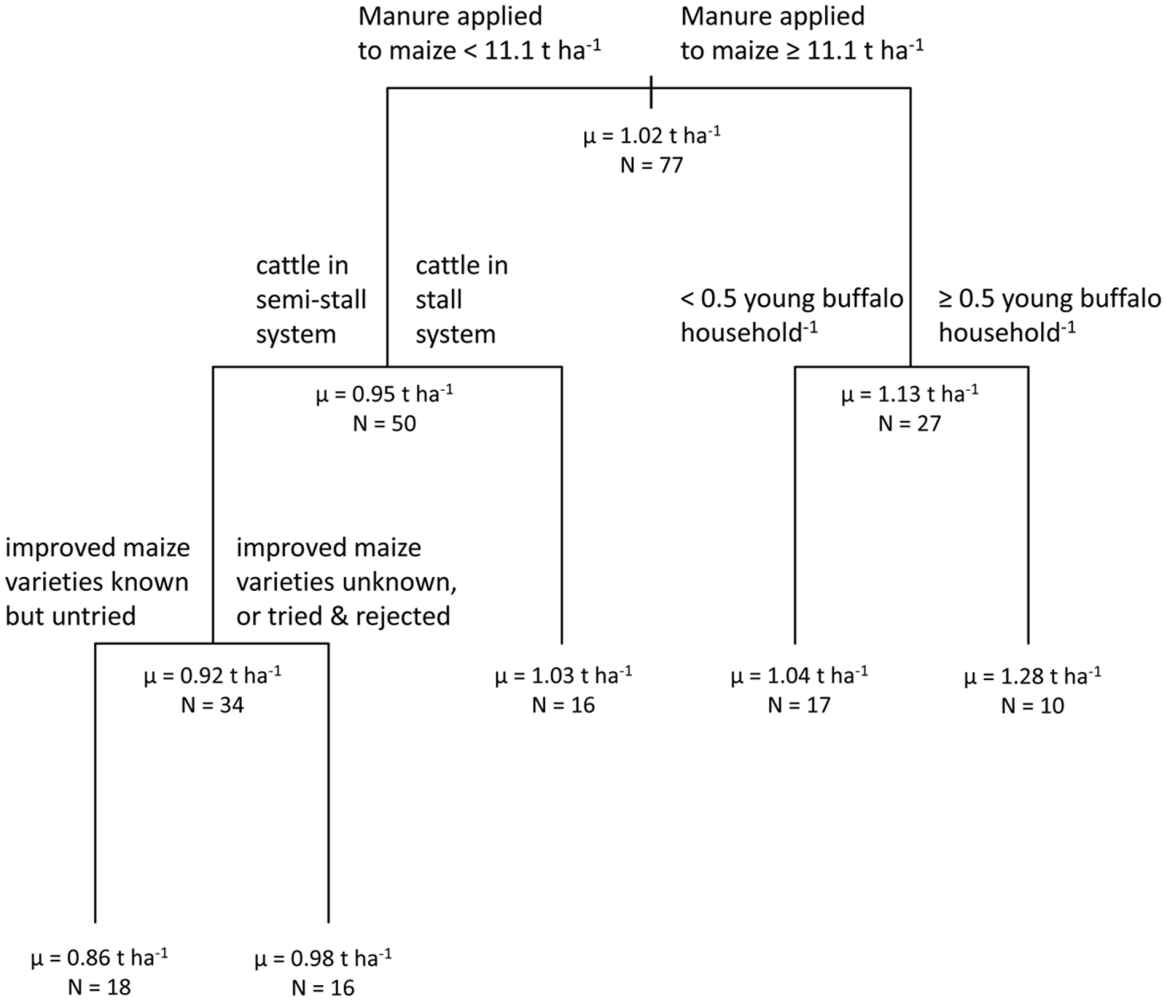
Maize yield. Classification and regression tree shows associations between the dependent variable ‘maize yield’ (within a maize-fingermillet relay system) and independent variables retained in the most parsimonious model.

Yield of maize in a maize-fingermillet relay system (Fig 5) was associated with agronomic and livestock management variables in a CART model with four branching nodes (R^2^ = 0.42). Manure application rate in maize was again the first conditioning variable in the tree, with the breakpoint in the primary node occurring at a manure application rate of 11.1 t ha^-1^. For those respondents reporting manure application rates lower than this benchmark, maize yields averaged 0.95 t ha^-1^, whereas for those respondents reporting higher application rates, maize yields averaged 1.13 t ha^-1^. Lower maize yields in subsequent branches were associated with management of cattle in a semi-stall-fed system, lack of adoption of improved maize varieties despite knowing about them, and low numbers of young buffalo.

#### Use of improved maize varieties

**Fig 6:**
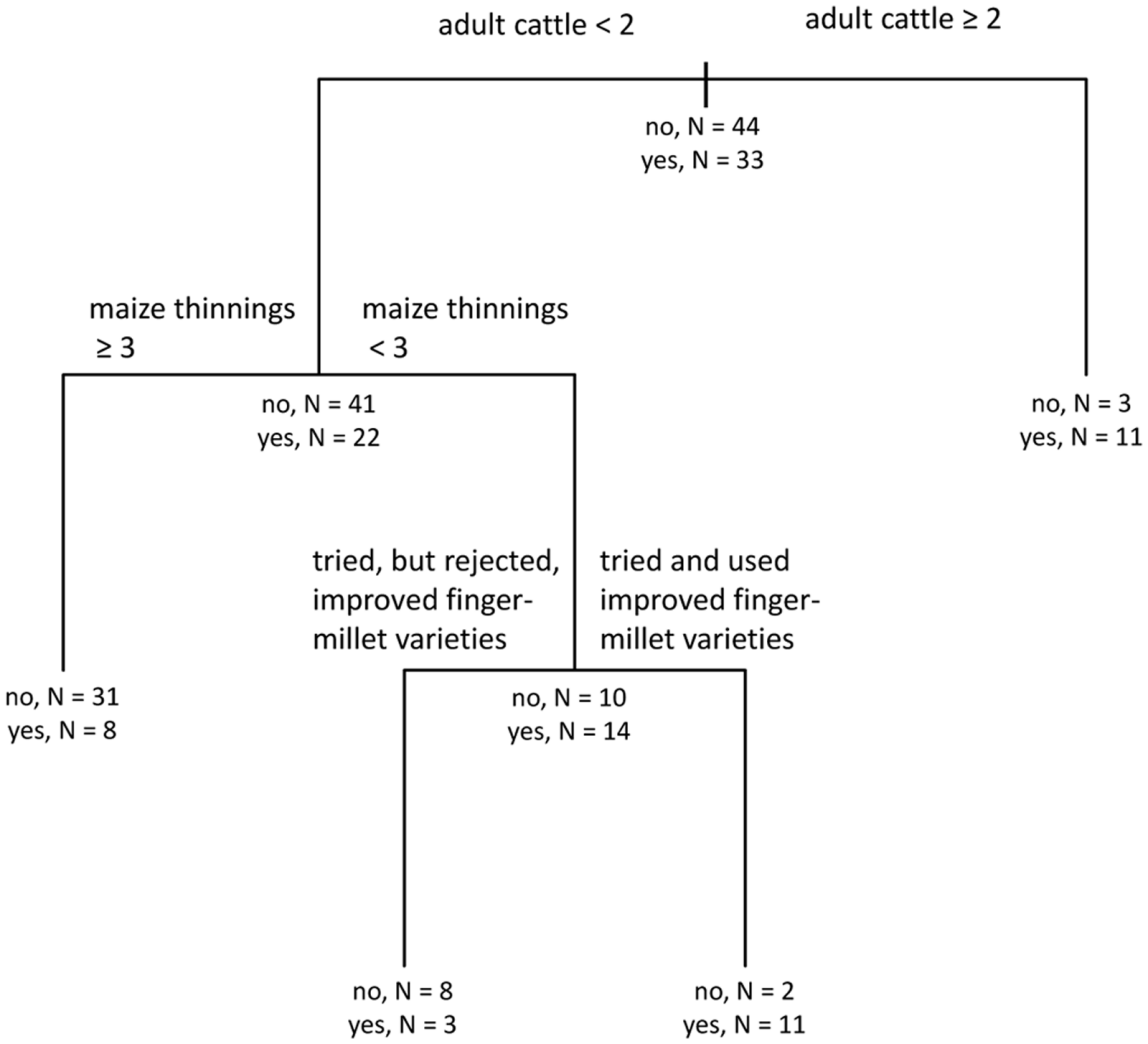
Adoption of improved maize cultivars. Classification and regression tree shows associations between the dependent variable ‘adoption of improved maize cultivars’ and independent variables retained in the most parsimonious model.

Three agronomic and livestock management variables (number of adult cattle, number of maize thinnings and adoption of improved fingermillet varieties) were retained in the most parsimonious CART model for adoption of improved maize varieties (Fig 6; R^2^ = 0.52). At the root node of the model, 43% of the respondents reported using improved open pollinated maize varieties. The first branch, at the root node, was conditioned by cattle ownership. Of those who reported owning less than 2 adult cattle (82% of the overall respondent pool), 65% did not use improved maize varieties. In contrast, of those who owned two or more adult cattle, only 21% did not use improved maize varieties. Among the group who owned less than two adult cattle, maize thinning practices were the next most influential variable. Respondents who reported three or more maize thinning operations during the growing season were unlikely to report using improved maize varieties (79% did not). In contrast, those who reported less than three maize thinnings were more likely to also report using improved maize varieties (58% used them). Within this latter group (those reporting less than three maize thinnings), those who tried but rejected improved fingermillet varieties were also likely to report that they rejected improved maize varieties (73% did not use them).

## Discussion

The retention of ‘variables of interest’ within our statistical models is not intended as a short list of proximal causes for food insecurity in the mid-hills of Nepal. Rather, we attempt to highlight dimensions of the problem that appear to be most closely associated with food security outcomes that are based on farm-level production, addressed here as household FSS. This is an approach that can be adapted to other mountain regions, and used to compare the relative importance of factors with potential impacts on FSS. Insights from these types of analysis can help define critical entry points for agricultural research and development.

At both levels of our nested analysis, variables related to crop and animal production practices showed prominent associations with dependent variables in the CART models. We interpret this as emphasizing the need for continued agricultural intensification: improvement of crop yields through use of improved cultivars and appropriate plant populations and boosting soil quality and fertility through addition of soil organic amendments in the form of animal manure. Land tenure (represented as Bari area) and socioeconomic issues (represented by literacy) made minor appearances, and labor did not show up in any of the most parsimonious models.

Evidence of the need to increase crop yields through adoption of improved crop cultivars and agricultural intensification supports the work of other research teams working in this area [4,10,16,22]. The information on animal husbandry practices associated with improved crop yields and variety adoption helps to highlight particularly useful features of crop-livestock integration. Larger animal holdings contribute to increased manure production, especially if animals are kept within stalls at least part of the time to concentrate manure for easier distribution. Greater rates of manure application not only support higher crop yields, but are necessary to meet the nutrient demands of higher yielding improved crop varieties, if no chemical fertilizer is applied.

Adoption of improved fingermillet varieties was much lower (14%) than use of improved maize varieties (43% overall). Only 3 varieties of fingermillet have been released by NARC to date. These varieties generally have comparatively longer crop duration and hence there may be producer reluctance to adopt these varieties out of concern for potential planting delays of subsequent winter crops. Our CART models indicated that, despite its low yields, fingermillet appears to make a major contribution to household FSS in the hills. Of the total maize growing areas of the hills, 80% are relayed with fingermillet [9,23]. Therefore, the development of improved fingermillet varieties with characteristics that are acceptable to farmers in the mid-hills should be an important breeding goal.

A related set of on-farm studies of the agricultural production practices of the survey population [23,24] support the central finding of this survey, that agronomic intensification and improved technical know-how can contribute greatly to crop yield (and thus household FSS) in this area. On-farm test plots showed benefits of improved crop cultivars, soil fertility and weed management, in comparison to surveyed yields [24,25]. Test yields, using improved crop varieties and recommended soil fertility and weed management practices, averaged 2.6 t ha^-1^ for maize and 1.4 t ha^-1^ for fingermillet. Planting improved maize varieties at recommended plant population densities in test plots further increased yield to 3.5 t ha^-1^. The traditional practice of overplanting maize, followed by repeated thinnings for animal fodder appears to be risky, and associated with production of land races rather than improved crop varieties. Possible reasons for this could range from lack of knowledge of ‘better-bet’ agronomy, to lower relative valued placed on maize grain production, to an overstocking of animal units.

Improved soil fertility and weed management also increased yields of farmer maize varieties: growing these varieties with recommended soil fertility practices produced 2.6 t ha^-1^ of grain under weed free conditions, and 1.7 t ha^-1^ under local weed management practices. These consist of one hand hoeing at 30–35 days after seeding, followed by one ‘earthing up’ operation (superficial soil disturbance forming ridges and furrows, in which the maize row becomes the ridge) prior to tasseling. Crop yield losses to weeds under this local system averaged 35% in both maize and fingermillet crops in test plots [24, 25].

The value of these related studies in interpreting the survey results is that they demonstrate, from controlled studies of farmer practices in comparison with current agronomic recommendations, that the correlative associations identified by the surveys are indicative of the considerable potential for increased productivity that remains to be realized by farmers in the survey region.

## Conclusions

Results of this work can be used to help improve the situation of villagers living in the mid-hills of Nepal by prioritizing technological and policy-level interventions. The data presented here underscore the importance of addressing yield gaps in ameliorating household FSS, especially in mountain and other less accessible areas where markets are comparatively weak and farm production strongly affects household welfare. Basic information and implementation of better-bet crop and livestock production practices appear to continue to play a central role in FSS outcomes in the surveyed region. Because there is solid knowledge of entry points for improving on-farm productivity, yet farmer adoption of these practices continues to lag, it is important to understand what factors are hindering technology transfer in this region. Certainly there are knowledge bottlenecks that are exacerbated by the provision of information in forms that many farmers do not find actionable or relevant. Another contributing factor is likely the lack of sufficient adaptation of management technologies to smallholder circumstances; participatory technology evaluations, iterative refinement, and domain targeting are approaches that are unevenly applied in the process of agricultural research for development in much of South Asia at present. Perhaps most fundamentally, farmers may have different incentives and decision processes that guide both their willingness and capacity to innovate and implement specific practices. Further work on improving the efficacy of technology transfer for key determinants of FSS appears to be urgently needed as a precursor to reducing hunger in the mid-hills of Nepal.

Integrated approaches to agricultural development are essential, but decision and intervention design ‘paralysis’ are not uncommon given the complexity of the contributing biophysical and socioeconomic factors. Data mining techniques such as those deployed in this analysis can help simplify the picture.

## Supporting Information

S1 Text Description and univariate statistics for variables included in farmer survey. (DOCX)

## References

[1] Foley JA, Ramankutty N, Brauman KA, Cassidy ES, Gerber JS, Johnston M, et al Solutions for a cultivated planet. Nature. 2011; 478: 337–342. doi: 10.1038/nature10452 21993620 10.1038/nature10452

[2] van Ittersum MK, Cassman KG, Grassini P, Wolf J, Tittonell P, Hochman Z. Yield gap analysis with local to global relevance--a review. Field Crops Res. 2013; 143: 4–17.

[3] Olmstead J. Feeding the world? Twelve years later, U.S. grain exports are up, so too is hunger. 2011 Dec 5 [cited 2/2/2015] In: Institute for Agriculture and Trade Policy. Available: http://www.iatp.org/documents/feeding-the-world.

[4] Gurung B, Gurung P. Addressing food scarcity in marginalized mountain environments—A participatory seed management initiative with women and men in eastern Nepal. Mtn Res Devel. 2002; 22: 240–247.

[5] Dame J, Nusser M. Food security in high mountain regions: agricultural production and the impact of food subsidies in Ladakh, Northern India. Food Security. 2011; 3: 179–194.

[6] Marquis G, Baldassarri T, Hofer T, Romeo R, Wolter P. FAO's Current Engagement in Sustainable Mountain Development. Mtn Res Devel. 2012; 32: 226–230.

[7] Maharjan KL, Joshi NP. Determinants of household food security in Nepal: A binary logistic regression analysis. J Mtn Sci. 2011; 8: 403–413.

[8] World Food Programme. WFP food security atlas of Nepal. U.N. World Food Programme. 2010 Jul 1 [cited 2/2/2015] In: World Food Programme. Available: http://www.wfp.org/content/nepal-food-security-atlas-2010. Accessed on 11/12/2013.

[9] Subedi KD. Maize and finger millet relay intercropping system in the hills of Nepal: Issues for sustainability. Proceedings of Sustainable Maize Production Systems for Nepal, Kathmandu, Nepal; 2001.

[10] Tiwari TP, Virk DS, Sinclair FL. Rapid gains in yield and adoption of new maize varieties for complex hillside environments through farmer participation I. Improving options through participatory varietal selection (PVS). Field Crops Res. 2009; 111: 137–143.

[11] Dahal BM, Nyborg I, Sitaula BK, Bajracharya RM. Agricultural intensification: food insecurity to income security in a mid-hill watershed of Nepal. Intl J Agric Sust. 2009; 7: 249–260.

[12] Gartaula H, Niehof A, Visser L. Shifting perceptions of food security and land in the context of labour out-migration in rural Nepal. Food Security. 2012; 4: 181–194.

[13] Tiwari TP, Brook RM, Sinclair EL. Implications of hill farmers' agronomic practices in Nepal for crop improvement in maize. Expt Agric. 2004; 40: 397–417.

[14] Pilbeam CJ, Mathema SB, Gregory PJ, Shakya PB. Soil fertility management in the mid-hills of Nepal: Practices and perceptions. Agric Human Val. 2005; 22: 243–258.

[15] Dawadi D, Sah S. Growth and yield of hybrid maize (*Zea mays* L.) in relation to planting density and nitrogen levels during winter season in Nepal. Trop Agric Res. 2012; 23: 218–227.

[16] Joshi KD, Devkota KP, Harris D, Khanal NP, Paudyal B, Sapkota A, et al Participatory research approaches rapidly improve household food security in Nepal and identify policy changes required for institutionalisation. Field Crops Res. 2012; 131: 40–48.

[17] Rea LM, Parker RA. Designing and conducting survey research. San Francisco, CA: Jossey-Bass Publishers; 1997.

[18] Breiman L, Friedman J, Olshen R, Stone C. Classification and Regression Trees. New York, NY: Chapman & Hall; 1984.

[19] De'ath G, Fabricius KE. Classification and regression trees: A powerful yet simple technique for ecological data analysis. Ecology. 2000; 81: 3178–3192.

[20] R Development Core Team. R: A language and environment for statistical computing. Vienna, Austria: R Foundation for Statistical Computing; 2009.

[21] Raut N, Sitaula BK, Aune JB, Bajracharya RM. Evolution and future direction of intensified agriculture in the central mid-hills of Nepal. Intl J Agric Sust. 2011; 9: 537–550.

[22] Venables WN, Ripley BD. Modern applied statistics with S. 4th Ed.: Springer, Berlin.; 2002.

[23] Subedi KD. Effect of leaf stripping, de-tasselling and topping of maize on the yield of maize and relay intercropped finger millet. Expt Agric. 1996; 32: 57–61.

[24] Karki T. B., Sah S. K., Thapa R. B., McDonald A. J., Davis A. S, Khadka Y. G.. Weeds and their effect on the performance of maize and fingermillet in the mid-hills of Nepal. Intl J Appl Sci Biotech. 2014a; 2: 275–278.

[25] Karki TB, Sah SK, Thapa RB, McDonald AJ, Davis AS. 2014b Plant density affects the productivity of maize-fingermillet systems in the mid-hills of Nepal. J Agric Allied Sci. 2014b; 3: 24–29.

