# Supplementary material for: Identifying Pathways for Improving Household Food Self-Sufficiency Outcomes in the Hills of Nepal
Source: PLoS One. 2015 Jun 5;10(6):e0127513. doi: 10.1371/journal.pone.0127513 (PMC4457816; doi:10.1371/journal.pone.0127513)
Supplement: S1 Text — (DOCX) [file pone.0127513.s001.docx]

**Appendix S1. Description and univariate statistics for variables included in farmer survey. For all variables, N=77.**

| **Variable name** | **Variable description (categorical levels in parentheses)** | **Dimension** | **Variable type** | **Mean** | **s.e.** | **Categorical level**  **1 2 3 4** | | | |
| --- | --- | --- | --- | --- | --- | --- | --- | --- | --- |
| cattle.adult | Number of adult cattle per household | Animal husbandry | quantitative | 0.99 | 0.07 |  |  |  |  |
| cattle.young | Number of young cattle per household | Animal husbandry | quantitative | 0.10 | 0.04 |  |  |  |  |
| cattle.mgt | Cattle management (1 = semistall, 2 = stall) | Animal husbandry | qualitative |  |  | 58 | 19 |  |  |
| buff.adult | Number of adult buffalo per household | Animal husbandry | quantitative | 1.14 | 0.05 |  |  |  |  |
| buff.young | Number of young buffalo per household | Animal husbandry | quantitative | 0.26 | 0.05 |  |  |  |  |
| buff.mgt | Buffalo management (1 = semistall, 2 = stall) | Animal husbandry | qualitative |  |  | 74 | 3 |  |  |
| goat.adult | Number of adult goats per household | Animal husbandry | quantitative | 1.40 | 0.09 |  |  |  |  |
| goat.young | Number of young goats per household | Animal husbandry | quantitative | 1.95 | 0.10 |  |  |  |  |
| goat.mgt | Goat management (1 = semistall, 2 = stall, 3 = extensive) | Animal husbandry | qualitative |  |  | 5 | 2 | 70 |  |
| maize.yield | Grain yield of sole-crop maize (t ha^-1^) | Crop production | quantitative | 1.78 | 0.04 |  |  |  |  |
| m.fm.yield | Grain yield of maize in maize-fingermillet relay system (t ha^-1^) | Crop production | quantitative | 1.02 | 0.02 |  |  |  |  |
| fm.yield | Grain yield of fingermillet in maize-fingermillet relay system (t ha^-1^) | Crop production | quantitative | 0.91 | 0.02 |  |  |  |  |
| sb.yield | Grain yield of sole-crop soybean (t ha^-1^) | Crop production | quantitative | 0.96 | 0.03 |  |  |  |  |
| w.yield | Grain yield of sole-crop wheat (t ha^-1^) | Crop production | quantitative | 1.18 | 0.03 |  |  |  |  |
| envir.index | Mean grain yield of all crops grown by respondent (t ha^-1^) | Crop production | quantitative | 1.18 | 0.02 |  |  |  |  |
| maizeyld.increase | How has maize grain yield changed in past 10 years? (1 = increase, 2 = same, 3 = decrease) | Crop production | qualitative |  |  | 8 | 66 | 3 |  |
| fert.use.trend | How has fertilizer used changed in past 10 years? (1 = increase, 2 = same, 3 = decrease) | Crop production | qualitative |  |  | 57 | 19 | 1 |  |
| fym.use.trend | How has farmyard manure used changed in past 10 years? (1 = increase, 2 = same, 3 = decrease) | Crop production | qualitative |  |  | 3 | 28 | 46 |  |
| fym.maize | Farmyard manure use in maize (t ha^-1^) | Crop production | quantitative | 17.2 | 0.6 |  |  |  |  |
| fym.rice | Farmyard manure use in rice (t ha^-1^) | Crop production | quantitative | 13.4 | 0.6 |  |  |  |  |
| fert.urea.rate | Fertilizer application rate for urea (t ha^-1^) | Crop production | quantitative | 1.5 | 0.08 |  |  |  |  |
| fert.DAP.rate | Fertilizer application rate for diammonium phosphate (t ha^-1^) | Crop production | quantitative | 0.93 | 0.03 |  |  |  |  |
| urea.split.maize | Is urea applied in a split application? (1 = yes, 2 = no) | Crop production | qualitative |  |  | 61 | 16 |  |  |
| split.times.dap.maize | Number of split applications of urea | Crop production | quantitative | 1.83 | 0.05 |  |  |  |  |
| impr.var.maize | Are improved varieties of maize used by household? (1 = yes, 2 = no) | Crop production | qualitative |  |  | 44 | 33 |  |  |
| no.maize.impr.why | If improved varieties of maize are not used, why? (1 = unknown, 2 = known but not tried, 3 = tried but rejected, 4 = NA, used) | Crop production | qualitative |  |  | 33 | 7 | 6 | 31 |
| no.fm.impr.var.why | If improved varieties of fingermillet are not used, why? (1 = unknown, 2 = known but not tried, 3 = tried but rejected) | Crop production | qualitative |  |  | 49 | 23 | 5 |  |
| sd.rate.maize | Seeding rate of maize (10^3^ seeds ha^-1^) | Crop production | quantitative | 35.6 | 0.60 |  |  |  |  |
| sd.rate.fm | Seeding rate of fingermillet (10^3^ seeds ha^-1^) | Crop production | quantitative | 16.9 | 0.53 |  |  |  |  |
| thin.times.maize | Number of times maize is thinned for forage | Crop production | quantitative | 2.6 | 0.06 |  |  |  |  |
| first.thin.maize.das | First thinning time in maize (days after sowing) | Crop production | quantitative | 30 | 0.14 |  |  |  |  |
| sec.thin.maize.das | Second thinning time in maize (days after sowing) | Crop production | quantitative | 36 | 0.52 |  |  |  |  |
| third.thin.maize.das | Third thinning time in maize (days after sowing) | Crop production | quantitative | 40 | 3 |  |  |  |  |
| weed.times.maize | Number of times weeds are removed in maize | Crop production | quantitative | 2.91 | 0.03 |  |  |  |  |
| nursery.fm | Type of fingermillet nursery management [1 = bari (unirrigated), 2 = khet (irrigated)] | Crop production | qualitative |  |  | 18 | 59 |  |  |
| nurs.weed.prob.where | Where weeds are a problem in fingermillet nursery [1 = bari (unirrigated), 2 = khet (irrigated)] | Crop production | qualitative |  |  | 72 | 5 |  |  |
| field.weed.problem | Where weeds are a problem in field crops [1 = bari (unirrigated), 2 = khet (irrigated)] | Crop production | qualitative |  |  | 29 | 48 |  |  |
| weed.forage | Are weeds used as forage, or removed to reduce competition with crop (1 = forage, 2 = reduce competition) | Crop production | qualitative |  |  | 36 | 41 |  |  |
| labor.avail | Is sufficient labor available to work the farm? (1 = yes, 2 = no) | Labor | qualitative |  |  | 13 | 64 |  |  |
| labor.wage.adult.male | Adult male wage (NC Rupees day^-1^) | Labor | quantitative | 461 | 5.3 |  |  |  |  |
| labor.wage.adult.female | Adult female wage (NC Rupees day^-1^) | Labor | quantitative | 164 | 3.2 |  |  |  |  |
| labor.wage.children | Child wage (NC Rupees day^-1^) | Labor | quantitative | 68 | 1.8 |  |  |  |  |
| labor.available.when.needed | Is sufficient labor available to work the farm at critical times? (1 = yes, 2 = no) | Labor | qualitative |  |  | 11 | 66 |  |  |
| Labor.days.m2 | Amount of labor required to manage crop land (person-hours m^-2^ growing season^-1^) | Labor | quantitative | 86 | 1.2 |  |  |  |  |
| labor.share.proportion | Proportion of labor that is shared with neighbors | Labor | quantitative | 0.59 | 0.02 |  |  |  |  |
| labor.hire.prop | Proportion of labor that is hired | Labor | quantitative | 0.61 | 0.02 |  |  |  |  |
| labor.exchange.foodgrain | Is labor exchanged for food grains? (1 = yes, 2 = no) | Labor | qualitative |  |  | 5 | 72 |  |  |
| altitude | Altitude of homestead (m above sea level) | Land tenure | quantitative | 1254 | 18 |  |  |  |  |
| khet.area.ha | Area of irrigated land (ha) | Land tenure | quantitative | 0.15 | 0.03 |  |  |  |  |
| khet.no | Number of parcels of irrigated land | Land tenure | quantitative | 1.77 | 0.12 |  |  |  |  |
| khet.dist.hr | Distance of irrigated land from home (hours) | Land tenure | quantitative | 0.96 | 0.03 |  |  |  |  |
| bari.area.ha | Area of un-irrigated land (ha) | Land tenure | quantitative | 0.13 | 0.02 |  |  |  |  |
| bari.no | Number of parcels of un-irrigated land | Land tenure | quantitative | 1.73 | 0.12 |  |  |  |  |
| bari.dist | Distance of un-irrigated land from home (hours) | Land tenure | quantitative | 1.04 | 0.04 |  |  |  |  |
| khar.area.ha | Pasture area (ha) | Land tenure | quantitative | 0.045 | 0.003 |  |  |  |  |
| khar.no | Number of parcels of pasture land | Land tenure | quantitative | 0.77 | 0.06 |  |  |  |  |
| khar.dist | Distance of pasture from home (hours) | Land tenure | quantitative | 1.16 | 0.12 |  |  |  |  |
| forest.area.ha | Forest area (ha) | Land tenure | quantitative | 0.017 | 0.005 |  |  |  |  |
| forest.no | Number of parcels of forest land | Land tenure | quantitative | 0.38 | 0.07 |  |  |  |  |
| forest.dist | Distance of forest parcels from home (hours) | Land tenure | quantitative | 0.25 | 0.06 |  |  |  |  |
| land.rent | Some land is rented out to others (1 = yes, 2 = no) | Land tenure | qualitative |  |  | 8 | 69 |  |  |
| rent.khet.area | Area of irrigated land that is rented (ha) | Land tenure | quantitative | 0.019 | 0.001 |  |  |  |  |
| rent.khet.no | Number of parcels of irrigated land that is rented | Land tenure | quantitative | 0.013 | 0.001 |  |  |  |  |
| rent.khet.time | Distance of rented irrigated land from home (hours) | Land tenure | quantitative | 0.026 | 0.002 |  |  |  |  |
| rent.bari.area | Area of un-irrigated land that is rented (ha) | Land tenure | quantitative | 0.011 | 0 |  |  |  |  |
| rent.bari.no | Number of parcels of un-irrigated land that is rented | Land tenure | quantitative | 0.21 | 0.06 |  |  |  |  |
| rent.bari.time | Distance of rented unirrigated land from home (hours) | Land tenure | quantitative | 0.19 | 0.05 |  |  |  |  |
| tenancy | Respondent owns all of land farmed by household (1 = yes, 2 = no) | Land tenure | qualitative |  |  | 13 | 64 |  |  |
| sharecrop.prop | Portion of land that is share-cropped (1 = all, 2 = none) | Land tenure | qualitative |  |  | 11 | 66 |  |  |
| share.input | Party responsible for providing inputs (1 = tenant, 2 = owner) | Land tenure | qualitative |  |  | 67 | 10 |  |  |
| share.crop | Crop share agreement (1 = none, 2 = half) | Land tenure | qualitative |  |  | 70 | 7 |  |  |
| land.area.change.ha | Change in land area farmed over past 10 years (ha) | Land tenure | quantitative | 0.11 | 0.01 |  |  |  |  |
| village | Village residency of respondent (1 = Lungaun, 2 = Pang, 3 = Patlekhet) | Socioeconomic | qualitative |  |  | 18 | 24 | 35 |  |
| gender | Gender of respondent (1 = male, 2 = female) | Socioeconomic | qualitative |  |  | 29 | 48 |  |  |
| literacy | Is respondent literate? ( 1 = yes, 2 = no) | Socioeconomic | qualitative |  |  | 42 | 35 |  |  |
| ethnicity | Ethnicity of respondent (1 = Brahmin, 2 = Chhetri, 3 = Magar) | Socioeconomic | qualitative |  |  | 27 | 41 | 9 |  |
| food.sufficiency | Degree of food sufficiency (1: < 6 months food supply produced on farm, 2: 6-12 months food supply produced on farm) | Socioeconomic | qualitative |  |  | 66 | 11 |  |  |
